# Pan-cancer and single-cell analysis reveal the prognostic value and immune response of NQO1

**DOI:** 10.3389/fcell.2023.1174535

**Published:** 2023-07-31

**Authors:** Liping Shen, Shan Jiang, Yu Yang, Hongli Yang, Yanchun Fang, Meng Tang, Rangteng Zhu, Jiaqin Xu, Hantao Jiang

**Affiliations:** ^1^ Department of Clinical Laboratory, Taizhou Hospital of Zhejiang Province Affiliated to Wenzhou Medical, Taizhou, Zhejiang, China; ^2^ Department of Radiology, Jining No. 1 People’s Hospital, Jining, Shandong, China; ^3^ Department of Orthopedics, Taizhou Hospital of Zhejiang Province Affiliated to Wenzhou Medical, Taizhou, Zhejiang, China; ^4^ Department of Pathology, The First Affiliated Hospital of Soochow University, Suzhou, Jiangsu, China; ^5^ Department of Ultrasonography, Taizhou Hospital of Zhejiang Province Affiliated to Wenzhou Medical, Taizhou, Zhejiang, China; ^6^ Department of Ultrasonography, Jining No. 1 People’s Hospital, Jining, Shandong, China

**Keywords:** *NQO1*, pan-cancer, immune, prognosis, therapy

## Abstract

**Background:** Overexpression of the *NAD(P)H: Quinone Oxidoreductase 1 (NQOI)* gene has been linked with tumor progression, aggressiveness, drug resistance, and poor patient prognosis. Most research has described the biological function of the *NQO1* in certain types and limited samples, but a comprehensive understanding of the *NQO1*’s function and clinical importance at the pan-cancer level is scarce. More research is needed to understand the role of *NQO1* in tumor infiltration, and immune checkpoint inhibitors in various cancers are needed.

**Methods:** The *NQO1* expression data for 33 types of pan-cancer and their association with the prognosis, pathologic stage, gender, immune cell infiltration, the tumor mutation burden, microsatellite instability, immune checkpoints, enrichment pathways, and the half-maximal inhibitory concentration (IC50) were downloaded from public databases.

**Results:** Our findings indicate that the *NQO1* gene was significantly upregulated in most cancer types. The Cox regression analysis showed that overexpression of the *NQO1* gene was related to poor OS in Glioma, uveal melanoma, head and neck squamous cell carcinoma, kidney renal papillary cell carcinoma, and adrenocortical carcinoma. *NQO1* mRNA expression positively correlated with infiltrating immune cells and checkpoint molecule levels. The single-cell analysis revealed a potential relationship between the *NQO1* mRNA expression levels and the infiltration of immune cells and stromal cells in bladder urothelial carcinoma, invasive breast carcinoma, and colorectal cancer. Conversely, a negative association was noted between various drugs (17-AAG, Lapatinib, Trametinib, PD-0325901) and the *NQO1* mRNA expression levels.

**Conclusion:** NQO1 expression was significantly associated with prognosis, immune infiltrates, and drug resistance in multiple cancer types. The inhibition of the *NQO1*-dependent signaling pathways may provide a promising strategy for developing new cancer-targeted therapies.

## 1 Introduction

Cancer imposes a significant health and economic burden on patients and society (Chukasemrat et al., 2021). The incidence and mortality from cancer are increasing worldwide (Huang et al., 2018). Treatment strategies, including surgery, radiation therapy, and cytotoxic chemotherapy, are now available to manage the disease (Chang et al., 2018). The success of these treatments varies widely in different cancers. Metastasis is one of the main factors leading to treatment failure. Cytotoxic chemotherapy has an important role in managing systemic disease. However, cancers can become resistant to cytotoxic chemotherapy limiting its efficacy (Zhang et al., 2018). Novel immunotherapies aimed at stimulating the immune system to fight cancer show promising results in cancer treatment. Nevertheless, the efficacy of these therapies depends on the presence of specific tumor biomarkers, the tumor immune infiltration, and the tumor mutation burden (Bahmani et al., 2021). Thus, innovative, more targeted therapeutic interventions are urgently needed for effective cancer control and management.

*NAD(P)H: Quinone Oxidoreductase 1 (NQO1)* is a flavoenzyme that can be upregulated by the transcription factor *nuclear factor erythroid 2-related factor 2 (NRF2)*, which plays important roles in cell detoxification, cancer cell growth, and immune response (Awadallah et al., 2008; Kasai et al., 2016). As a result, it is considered a potential target for developing novel cancer therapies. *NQO1* works by catalyzing the mandatory two-electron reduction of large amounts of quinone to hydroquinone (Ross et al., 2000). This process avoids the one-electron reduction, which produces toxic semi-quinone radicals and reactive oxygen species (ROS) (Bianchet et al., 2008; Talalay and Dinkova-Kostova, 2004). The *NQO1*-directed reduction can also bioactivate certain antitumor quinones by forming unstable hydroquinone, which can alkylate DNA and produce large amounts of ROS through redox cycling (Siegel et al., 2012). *NQO1* can also mediate cell detoxification by reducing the quinone-imine metabolites and other drug-derived quinone-like species that can lead to adverse drug reactions (ADRs) (den Braver-Sewradj et al., 2018; Vredenburg et al., 2014). In addition, *NQO1* is also involved in other important biological processes, including stabilizing vital regulatory proteins under stress (Dinkova-Kostova and Talalay, 2010). Numerous studies have shown a significantly higher *NQO1* expression in many tumors, including breast (Bentle et al., 2007; Marin et al., 1997; Yang et al., 2014), lung (Bey et al., 2007; Cui et al., 2014; Li et al., 2015), prostate (Dong et al., 2010), head and neck cancer (Li et al., 2016), and gastrointestinal carcinomas (Lin et al., 2014), when compared to normal tissue. Furthermore, *NQO1* expression was also closely related to tumor progression, aggressiveness, resistance to chemotherapy, and poor patient outcomes (Hirose et al., 2021; Park et al., 2019; Xu et al., 2022). While most research has described the biological function of *NQO1* in certain types and limited samples, a comprehensive understanding of the *NQO1*’s function and clinical importance at the pan-cancer level is scarce. More research is needed to understand the role of *NQO1* in tumor infiltration, and immune checkpoint inhibitors in various cancers are needed.

This study aimed to systematically describe the immunological aspects and prognostic value of *NQO1* expression via a pan-caner and single-cell analysis. In order to achieve this aim, we collected data from different databases to investigate the role of *NQO1* on the immune response in various cancers. Subsequently, we evaluated the correlations between the *NQO1* expression and the tumor mutation burden, microsatellite instability, immune infiltration levels, and various immune-related genes in multiple cancer types. We then examined the biological functions and pathways of *NQO1* using Gene ontology (GO) analysis. Single-cell sequencing analyses were used to compare the *NQO1* expression levels between immune and stromal cells. Finally, the Genomics of Drug Sensitivity in Cancer (GDSC) and Cancer Therapeutics Response Portal (CTRP) datasets were used to evaluate the impact of *NQO1* expression on the response to various anticancer drugs. The outcomes of this study could be used to guide the development of new immunotherapies that target the *NQO1* gene.

## 2 Materials and methods

### 2.1 NQO1 expression pattern in human pan-cancer

To analyze the expression level of NQO1 in the pan-cancer, the ribonucleic acid (RNA) sequencing data and clinical follow-up information of 33 different types of cancers were extracted from the Cancer Genome Atlas (TCGA) database first. Further, log2 (x+0.001) transformation was performed for each expression value. Finally, we also excluded those cancers with less than three samples in a single cancer species and finally obtained the expression data for 34 cancer species. The RNA sequences of 31 types of normal tissues were retrieved from the Genotype-Tissue Expression (GTEx) dataset (https://www.genome.gov/Funded-Programs-Projects/Genotype-Tissue-Expression-Project). The cell line data were downloaded from the Human Protein Atlas (HPA) datasets (https://www.proteinatlas.org/) and the Cancer Cell Line Encyclopedia (CCLE) (https://sites.broadinstitute.org/ccle/). Methylation level analysis was obtained online through the Gene Set Cancer Analysis database (http://bioinfo.life.hust.edu.cn/GSCA/#/mutation). The abbreviations used for the pan cancers extracted from the databases are defined in Table 1.

**TABLE 1:** Abbreviations for the cancers searched in this study.

Abbreviations	Full name
ACC	Adrenocortical carcinoma
ALL	Acute lymphoeytic leukemia
BLCA	Bladder Urothelial Carcinoma
BRCA	Invasive breast carcinoma
CESC	Cervical squamous cell carcinoma and endocervical adenocarcinoma
CHOL	Cholangiocarcinoma
COAD	Colon adenocarcinoma
COADREAD	Colon adenocarcinoma/Rectum adenocarcinoma Esophageal carcinoma
CRC	Colorectal cancer
DLBC	Lymphoid Neoplasm Diffuse Large B-cell Lymphoma
ESCA	Esophageal carcinoma
FPPP	FFPE Pilot Phase II
GBM	Glioblastoma multiforme
GBMLGG	Glioma
HNSC	Head and Neck squamous cell carcinoma
KICH	Kidney Chromophobe
KIPAN	Pan-kidney cohort (KICH+KIRC+KIRP)
KIRC	Kidney renal clear cell carcinoma
KIRP	Kidney renal papillary cell carcinoma
LAML	Acute Myeloid Leukemia
LGG	Brain Lower Grade Glioma
LIHC	Liver hepatocellular carcinoma
LUAD	Lung adenocarcinoma
LUSC	Lung squamous cell carcinoma
MESO	Mesothelioma
OV	Ovarian serous cystadenocarcinoma
PAAD	Pancreatic adenocarcinoma
PCPG	Pheochromocytoma and Paraganglioma
PRAD	Prostate adenocarcinoma
READ	Rectum adenocarcinoma
SARC	Sarcoma
SKCM	Skin Cutaneous Melanoma
STAD	Stomach adenocarcinoma
STES	Stomach and Esophageal carcinoma
TGCT	Testicular Germ Cell Tumors
THCA	Thyroid carcinoma
THYM	Thymoma
UCEC	Uterine Corpus Endometrial Carcinoma
UCS	Uterine Carcinosarcoma
UVM	Uveal Melanoma
WT	Wilms Tumor

### 2.2 Immunohistochemistry (IHC) staining

To evaluate differences in NQO1 expression at the protein level, IHC images of NQO1 protein expression in normal and tumor tissues were downloaded from The Human Protein Atlas (HPA). In addition, the NQO1 expression data for breast carcinoma, ovarian serous cystadenocarcinoma, and pancreatic adenocarcinoma were verified by pathological tissue obtained from the Department of Pathology of the First Affiliated Hospital of Soochow University. Moreover, the IHC images of NQO1 protein subcellular localization were downloaded from the HPA database. The antibody of the NQO1 protein was HPA0077308.

### 2.3 Evaluation of the *NQO1* expression levels in relation to the tumor mutation burden, microsatellite instability, and genetic alterations of *NQO1*

To explore the association between *NQO1* mutations with gene expression levels, we analyzed the correlations of the *NQO1* mRNA expression levels with the tumor mutation burden and microsatellite instability. The relationship between *NQO1* mRNA expression and immune checkpoints genes, the tumor mutation burden, and microsatellite instability in the tumor microenvironment (TME) was explored via the SangerBox website (http://sangerbox.com/Tool). Firstly, the expression data of the *NQO1* gene downloaded from the TCGA database were screened for samples with sample sources of Primary Blood-Derived Cancer - Peripheral Blood, P*r*imary Tumor. We also downloaded the Simple Nucleotide Variation dataset of level 4 of all TCGA samples processed by MuTect2 software (Beroukhim et al., 2010) from GDC (https://portal.gdc.cancer.gov/). We calculated the Tumor mutation burden of each tumor with the tumor mutation burden function of the R package maftools (version 2.8.05). We obtained the microsatellite instability score for each tumor from a previous study (Bonneville et al., 2017). We integrated the tumor mutation burden and microsatellite instability of the samples with the gene expression data separately, and further log2 (x+0.001) transformation was performed for each expression value. The correlation coefficients of the tumor mutation burden and microsatellite instability with the expression of *NQO1* mRNA were calculated separately. The *NQO1* gene mutation frequency and copy number alteration were analyzed for all the datasets gathered in the TCGA pan-cancer atlas studies using the cBioPortal (version: 3.6.20) (https://www.cbioportal.org/).

### 2.4 Tumor immune estimation resource

To further explore the *NQO1* correlation with immune cell infiltration in pan-cancer, we downloaded the expression data of the *NQO1* gene from the TCGA database in each sample. Further, we screened the samples from Primary Blood-Derived Cancer - Peripheral Blood (TCGA-LAML), Primary Tumor, and TCGA-SKCM metastatic samples. In addition, we extracted the gene expression profiles of each tumor separately and mapped the expression profiles to GeneSymbol using the R package IOBR (version 0.99.9) (Zeng et al., 2021) with the timer (Li et al., 2017) and deconvo_epic method (Racle et al., 2017) and deconvo_mcpcounter method (Becht et al., 2016) We then reassessed each patient’s cancer-associated fibroblasts (CAFs), stromal cells and immune cells in each tumor based on its gene expression. The expression of two types of immune checkpoint genes (Inhibitory (24), and Stimulatory (36)) was obtained from the TCGA database. The correlation of immune checkpoints genes with *NQO1*was evaluated using the Spearman correlation analysis.

### 2.5 Single-cell sequencing analysis

To further explore the association of cell types and *NQO1* in the microenvironment of specific types of cancer cells for study, we analyzed the *NQO1* mRNA expression levels on immune cells in various cancer types using TISCH. TISCH (http://tisch1.comp-genomics.org/) is a standardized analysis tool used to explore cellular heterogeneity in the tumor microenvironment (TME) at both single-cell and annotated cluster levels. TISCH employs MAESTRO v1.1.0 as the standard analysis process for all collected datasets, which includes quality control, batch effect removal, cell clustering, differential expression analysis, cell type labeling, and malignant cell classification (Wang et al., 2020). Raw counts and Transcripts Per Million (TPM) tables were input into this standardization workflow. Cell quality was assessed based on two metrics-total counts per cell (Unique Molecular Identifier, UMI) and the number of genes detected per cell ber. Low-quality cells with a library size below 1,000 or a detected number of genes below 500 were filtered out (Sun et al., 2021).

### 2.6 *NQO1*-related gene enrichment and diseases analysis

A protein-to-protein interaction (PPI) network was built using the STRING online website (https://string-db.org/) and Cytoscape software version 3.7.1 (The Cytoscape Consortium, San Diego, CA, United States). The *NQO1*-related proteins were retrieved from the STRING online website. A functional annotation chart data was created by uploading the gene lists to the Database for Annotation, Visualization, and Integrated Discovery (DAVID) (https://david.ncifcrf.gov/). The DisGeNET database (http://www.disgenet.org) on the NetworkAnalyst website was used to search for NQO1-related diseases.

### 2.7 Prognostic analysis

The connection between the *NQO1* expression levels and outcomes, including overall survival (OS), disease-specific survival (DSS), disease-free interval (DFI), and progression-free interval (PFI) in 33 types of cancer, was examined using forest plots. The hazard ratios (HRs) and 95% confidence intervals (95%CI) of *NQO1* on outcomes were calculated using univariate Cox regression analysis. After separating patients into high and low *NQO1* expression groups through the optimal cut-off value, the Kaplan–Meier method was used to create the survival curves of patients in each cancer type.

### 2.8 Drug sensitivity analysis

The GDSC and the CTRP databases were used to evaluate the underlying response to chemotherapy to the various cancers extracted from GSCA (Gene Set Cancer Analysis) (http://bioinfo.life.hust.edu.cn/). We collected the half-maximal inhibitory concentration (IC50) of 265 small molecules in 860 cell lines and its corresponding mRNA gene expression from GDSC. We collected the IC50 of 481 small molecules in 1001 cell lines and its corresponding mRNA gene expression from CTRP. The mRNA expression data and drug sensitivity data were merged. Pearson correlation analysis was performed to evaluate the correlation between gene mRNA expression and drug IC50. False discovery rate (FDR) adjusted the *P*-value.

### 2.9 Ethical considerations

The study was conducted according to the guidelines of the Declaration of Helsinki and approved by the ethics committee of Taizhou Hospital of Zhejiang Province (K20230202).

### 2.10 Statistical analysis

The Student’s *t*-test was used to compare the *NQO1* mRNA expression levels between cancerous and normal tissues. The Kruskal-Wallis test was used to compare the *NQO1* mRNA expression levels between different groups, tumor stages, subtypes, and gender. Spearman correlation analysis was used to compare the *NQO1* mRNA expression levels with the tumor mutation burden, microsatellite instability, and checkpoint-related genes, and heatmaps were used to visualize the correlations. Univariate Cox regression analysis was used to assess the impact of the *NQO1* mRNA expression levels on prognosis. Kaplan–Meier method was used to create the survival curves of patients. For all statistical tests, a *p*-value below 0.05 was deemed statistically significant.

## 3 Results

### 3.1 Expression of NQO1 in pan-cancer

**FIGURE 1:**
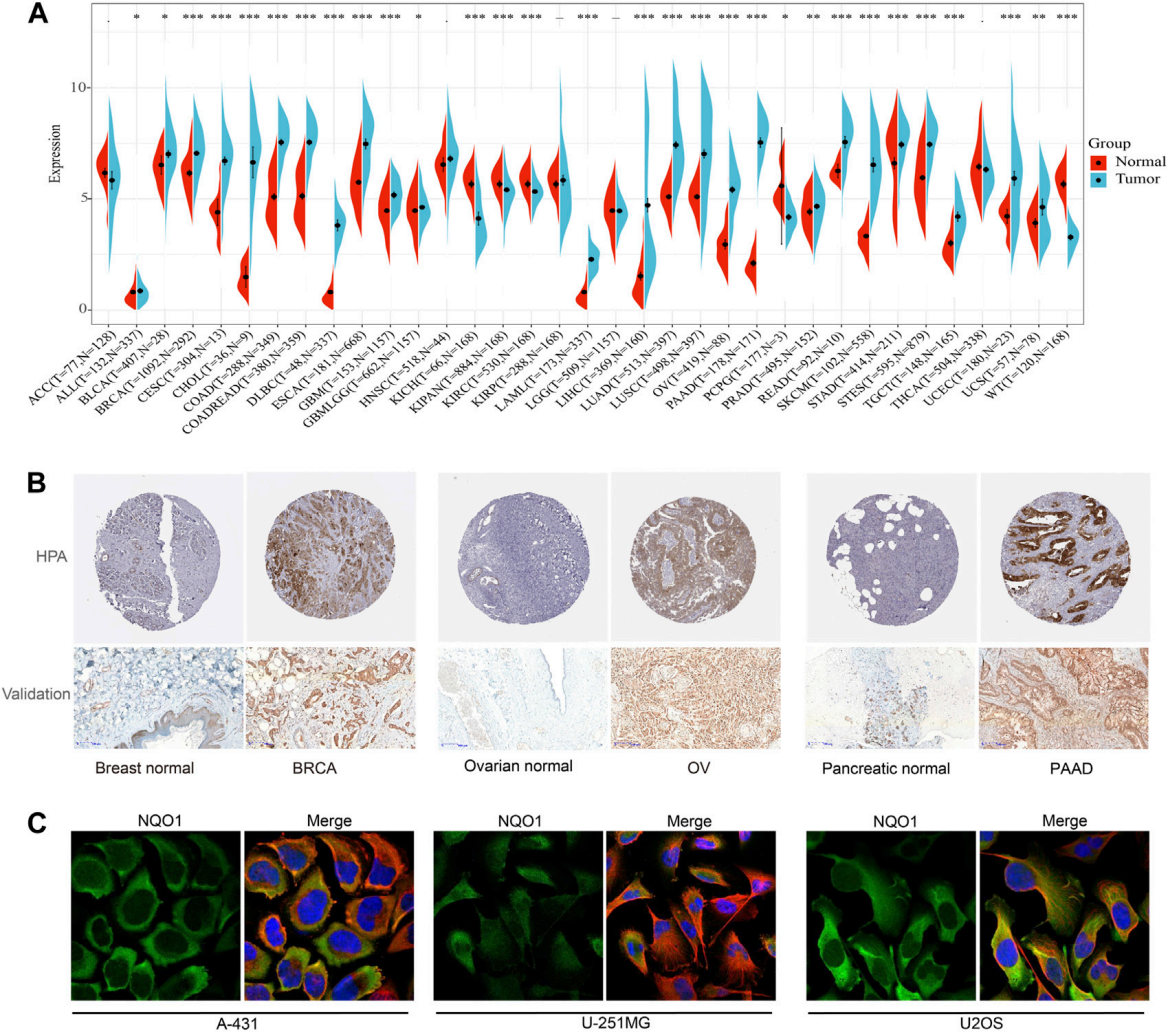
Expression level of *NAD(P)H: Quinone Oxidoreductase 1 (NQO1)* mRNA in the human pan-cancer. **(A)** The *NQO1* mRNA expression levels in cancerous (blue bar) and normal (red bar) tissues according to the data extracted from the Cancer Genome Atlas (TCGA) and the Genotype-Tissue Expression (GTEx) databases. **(B)** Immunohistochemistry staining of the NQO1 protein expression levels between normal and cancerous tissue, including breast, ovarian, and pancreatic. **(C)** Immunohistochemistry staining of NQO1 protein of A-431 (epidermoid tumor cell line), U-251MG (Malignant glioma cell line), and U2OS (osteosarcoma cell line).

We first evaluated the *NQO1* mRNA expression levels in 31 normal tissues extracted from the GTEx. The top five *NQO1*-enriched normal tissues in this database were the stomach, thyroid gland, esophagus, adipose tissue, and urinary bladder (Supplementary Figure S1A). Compared to normal tissue, *NQO1* mRNA is highly expressed in most tumors, except for kidney chromophobe, pan-kidney cohort, kidney renal papillary cell carcinoma, pheochromocytoma and paraganglioma, and wilms tumor (Figure 1A). Unlike *NQO1*, *NRF2* was differentially expressed from controls in adrenocortical carcinoma, kidney renal papillary cell carcinoma, brain lower grade glioma, and thyroid carcinoma, but not in cervical squamous cell carcinoma and endocervical adenocarcinoma, colon adenocarcinoma, kidney renal papillary cell carcinoma, pheochromocytoma and paraganglioma, and rectum adenocarcinoma. *NRF2* was underexpressed in bladder urothelial carcinoma, lung adenocarcinoma, ovarian serous cystadenocarcinoma, prostate adenocarcinoma, skin cutaneous melanoma, testicular germ cell tumors, uterine corpus endometrial carcinoma, and uterine carcinosarcoma. Both *NQO1* and *NRF2* did not differ significantly from the controls in head and neck squamous cell carcinoma. Similarly to *NQO1*, *NRF2* was elevated in acute lymphoeytic leukemia, cholangiocarcinoma, lymphoid neoplasm diffuse large B-cell lymphoma, esophageal carcinoma, glioblastoma multiforme, glioma, acute myeloid leukemia, brain lower grade glioma, liver hepatocellular carcinoma, pancreatic adenocarcinoma, stomach adenocarcinoma, stomach and esophageal carcinoma; and decreased in kidney chromophobe, kidney renal clear cell carcinoma, kidney renal clear cell carcinoma, kidney renal papillary cell carcinoma, and wilms tumor (Supplementary Figure S2A). In addition, the *NQO1* methylation levels were significantly lower in uterine carcinosarcoma, liver hepatocellular carcinoma, pancreatic adenocarcinoma, bladder urothelial carcinoma, bladder urothelial carcinoma, and lung squamous cell carcinoma (Supplementary Figure S2B)*.* Subsequently, we assessed the *NQO1* mRNA expression levels in 32 tumor cell lines extracted from the CCLE and 69 tumor cell lines extracted from the HPA databases. The results showed that skin cutaneous melanoma, skin cutaneous melanoma, brain lower grade glioma, lung squamous cell carcinoma, mesothelioma, and lung adenocarcinoma had high expression levels of *NQO1* mRNA (Supplementary Figure S1B). The top five *NQO1* mRNA-enriched cell lines in the HPA database were OE19 (Esophageal adenocarcinoma cell line), SiHa (Cervical cancer cell line), A549 (Alveolar cell carcinoma cell line), WM-115 (Melanoma cell line), and LHCN-M2 (Prostate cancer cell line) (Supplementary Figure S1C). Furthermore, to evaluate the NQO1 expression at the protein level, we analyzed the IHC results provided by the HPA database (Figure 1B; Supplementary Figure S3A). The protein expression levels were consistent with the mRNA levels. The NQO1 protein expression level in invasive breast carcinoma, ovarian serous cystadenocarcinoma, and pancreatic adenocarcinoma was verified by pathological tissue from the Department of Pathology of the First Affiliated Hospital of Soochow University (Figure 1B). To further analyze the NQO1 protein subcellular localization, we analyzed the expression profiles in the HPA database. The result showed that the NQO1 protein was localized to the cytosol of A-431 (epidermoid tumor cell line), U-251MG (Malignant glioma cell line), and U2OS (osteosarcoma cell line) (Figure 1C).

### 3.2 Association between the *NQO1* mRNA expression levels with the pathological stage, subtypes, and gender for various cancers

**FIGURE 2:**
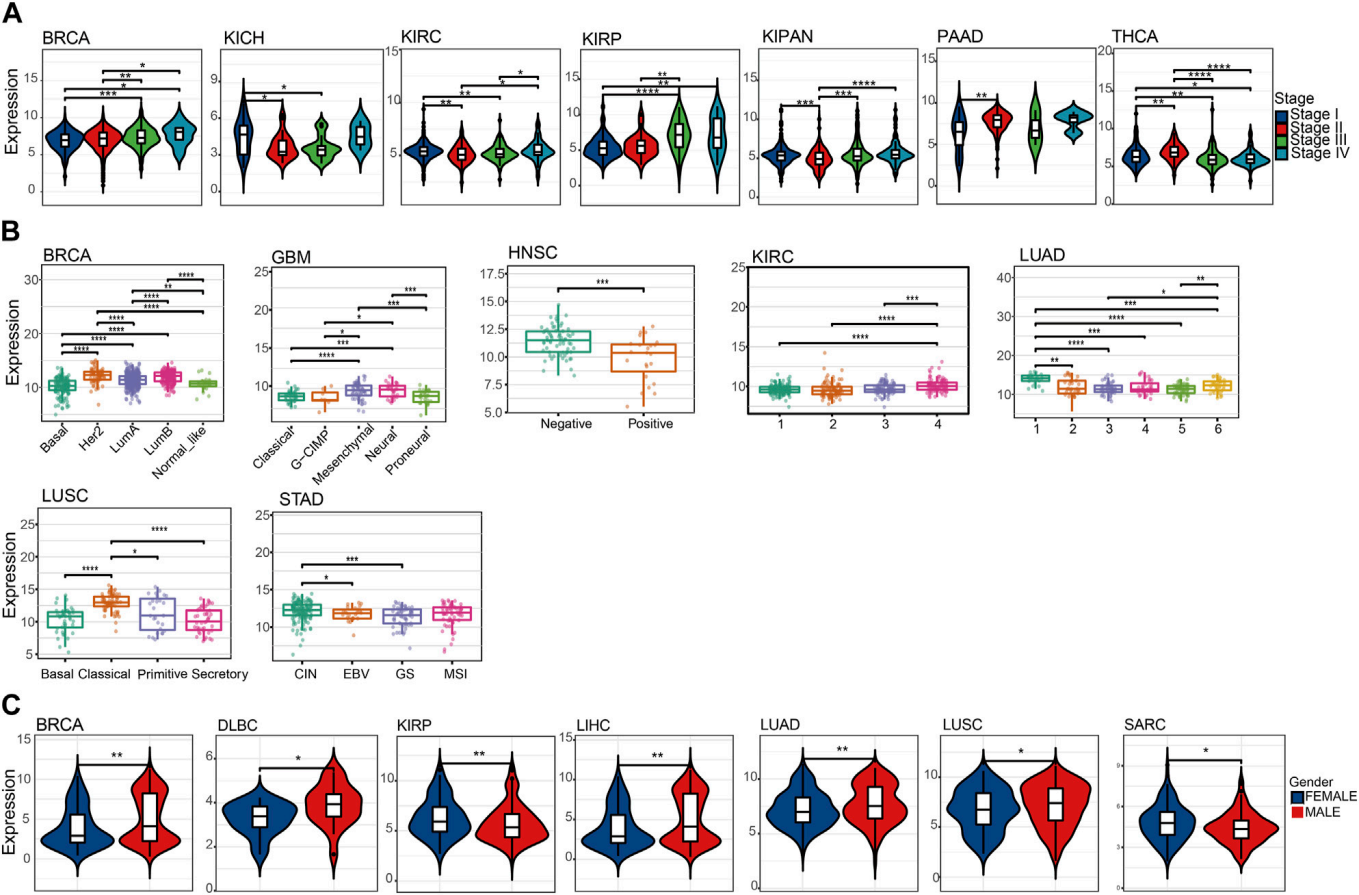
Association between the *NQO1* expression levels according to the pathological stages, subtypes, and gender for pan-cancer. **(A)** The *NQO1* mRNA expression levels concerning the pathological stages, including invasive breast carcinoma (BRCA), kidney chromophobe (KICH), kidney renal clear cell carcinoma (KIRC), kidney renal papillary cell carcinoma (KIRP), pan-kidney cohort (KICH+KIRC+KIRP) (KIPAN), pancreatic adenocarcinoma (PAAD) and thyroid carcinoma (THCA). **(B)** The *NQO1* mRNA expression levels with the subtypes, including BRCA, glioblastoma multiforme (GBM), head and neck squamous cell carcinoma (HNSC), KIRC, lung adenocarcinoma (LUAD), lung squamous cell carcinoma (LUSC) and stomach adenocarcinoma (STAD). **(C)** Comparison of the *NQO1* mRNA expression levels in males and females, including BRCA, lymphoid neoplasm diffuse large B-cell lymphoma (DLBC), KIRP, liver hepatocellular carcinoma (LIHC), LUAD, LUSC, and Sarcoma (SARC). *p < 0.05; **p < 0.01; ***p < 0 .001.

To better understand the significance of NQO1 in tumorigenesis, progression, and prognosis, we analyzed the relationship between the *NQO1* mRNA expression levels and the pathological stages in multiple cancers. The *NQO1* mRNA expression levels were significantly associated with the pathological stages in pancreatic adenocarcinoma, thyroid carcinoma, pan-kidney cohort, invasive breast carcinoma, and kidney chromophobe. Higher expression levels of *NQO1* mRNA were correlated with more advanced pathological stages in kidney renal papillary cell carcinoma and invasive breast carcinoma; In kidney chromophobe, kidney renal clear cell carcinoma, and pan-kidney cohort, *NQO1* mRNA levels are lowest at SatgeⅡ. In contrast, invasive breast carcinoma and thyroid carcinoma are the highest at stage Ⅱ (Figure 2A). We also analyzed the relationship between the *NQO1* mRNA expression levels and the tumor subtypes. *NQO1* mRNA was mostly expressed in Her2, followed by LumB and basal invasive breast carcinoma. In Glioblastoma multiforme, *NQO1* mRNA expression was highest in mesenchymal and neural. *NQO1* mRNA expression was highest in type 1 lung adenocarcinoma. The classical type has the highest *NQO1* mRNA expression in lung squamous cell carcinoma. The chromosome instability (CIN) type had the highest stomach adenocarcinoma’s *NQO1* mRNA expression (Figure 2B). Besides, *NQO1* mRNA expression was higher in males withinvasive breast carcinoma, lymphoid neoplasm diffuse large B-cell lymphoma, liver hepatocellular carcinoma, lung adenocarcinoma, and lung squamous cell carcinomathan in females. Conversely, the *NQO1* mRNA expression was lower in males with kidney renal papillary cell carcinoma and sarcoma (Figure 2C). However, *NQO1* did not differ between gender in control groups (Supplementary Figure S2C). Besides, *NQO1* mRNA expression levels were positively associated with the age of patients in the pan-kidney cohort, brain lower grade glioma, and glioma. In contrast, *NQO1* mRNA expression levels were negatively associated with the patients’ age in ovarian serous cystadenocarcinoma, lung squamous cell carcinoma, and thyroid carcinoma (Supplementary Figure S3B).

### 3.3 Relationship between the *NQO1* mRNA expression and the tumor mutation burden, microsatellite instability, and genetic alterations of *NQO1*

**FIGURE 3:**
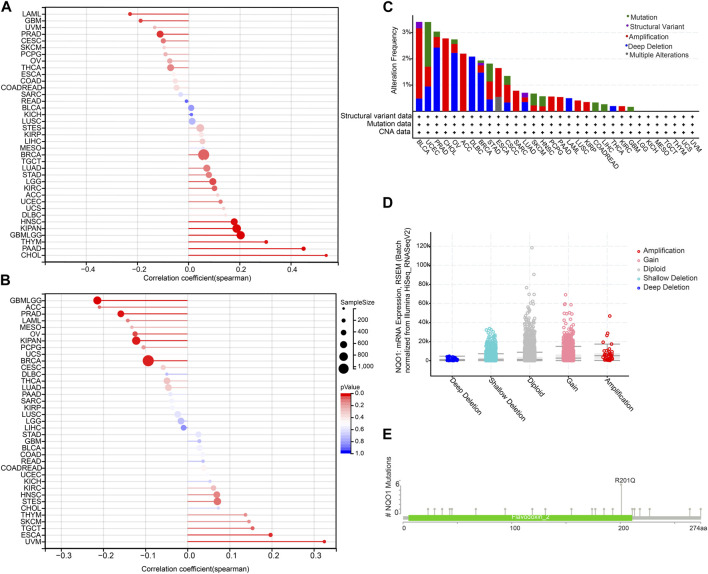
Correlation of *NQO1* mRNA expression with the tumor mutation burden (TMB), microsatellite instability (MSI), and genetic alterations of *NQO1*. Correlation between the *NQO1* mRNA expression levels with TMB **(A)** and MSI **(B)** for different types of cancer. The size of the dots in the figure represents the size of the correlation coefficient, and the different colors represent the significance of the *P*-value. The *NQO1* genetic alterations in pan-cancer according to the mutation site **(C)** and the copy number variants **(D)**. **(E)** the primary *NQO1* genetic alterations in all cancers.

The tumor mutation burden refers to the number of mutations present in tumor cells and is usually calculated by measuring the frequency of mutations in certain genes. In contrast, microsatellite instability refers to the altered number of recurrences of certain microsatellite sequences in cancer tissue compared to normal tissue. NQO1 is a redox enzyme that is involved in regulating the redox balance of the intracellular environment and has a protective effect against damage to cellular DNA. Therefore *NQO1* may be involved in the repair process of tumor DNA; thus, its expression may affect the levels of the tumor mutation burden and microsatellite instability (Preethi et al., 2022). In view of this, we decided to analyze the correlation between the *NQO1* mRNA expression levels, the tumor mutation burden, and microsatellite instability. The tumor mutation burden was positively correlated with the *NQO1* expression levels in cholangiocarcinoma, pancreatic adenocarcinoma, thymoma, glioma, kidney renal papillary cell carcinoma, head and neck squamous cell carcinoma, and negatively correlated in glioblastoma multiforme, acute myeloid leukemia, and prostate adenocarcinoma (Figure 3A). Microsatellite instability was positively correlated with the *NQO1* mRNA expression levels in esophageal carcinoma and uveal melanoma and negatively correlated with uveal melanoma, invasive breast carcinoma, pan-kidney cohort, prostate adenocarcinoma, and ovarian serous cystadenocarcinoma (Figure 3B). The cBioPortal (TCGA, Pan-Cancer Atlas) database was used to evaluate the pan-cancer alterations of the *NQO1* gene. Bladder urothelial carcinoma had the highest frequency (approximately 3%) of *NQO1* alteration (Figure 3C). Other tumors, including acute myeloid leukemia and diffuse large B-cell lymphoma had lower *NQO1* mutation frequency. Mutation sites were detected between amino acids 0 and 274, including 23 missense mutations, three fusions, one in-frame deletion, and one splice (Figure 3D). The most common genetic alterations were gained and shallow deletion (Figure 3E).

### 3.4 Correlation between *NQO1* mRNA expression level and cancer immunity

**FIGURE 4:**
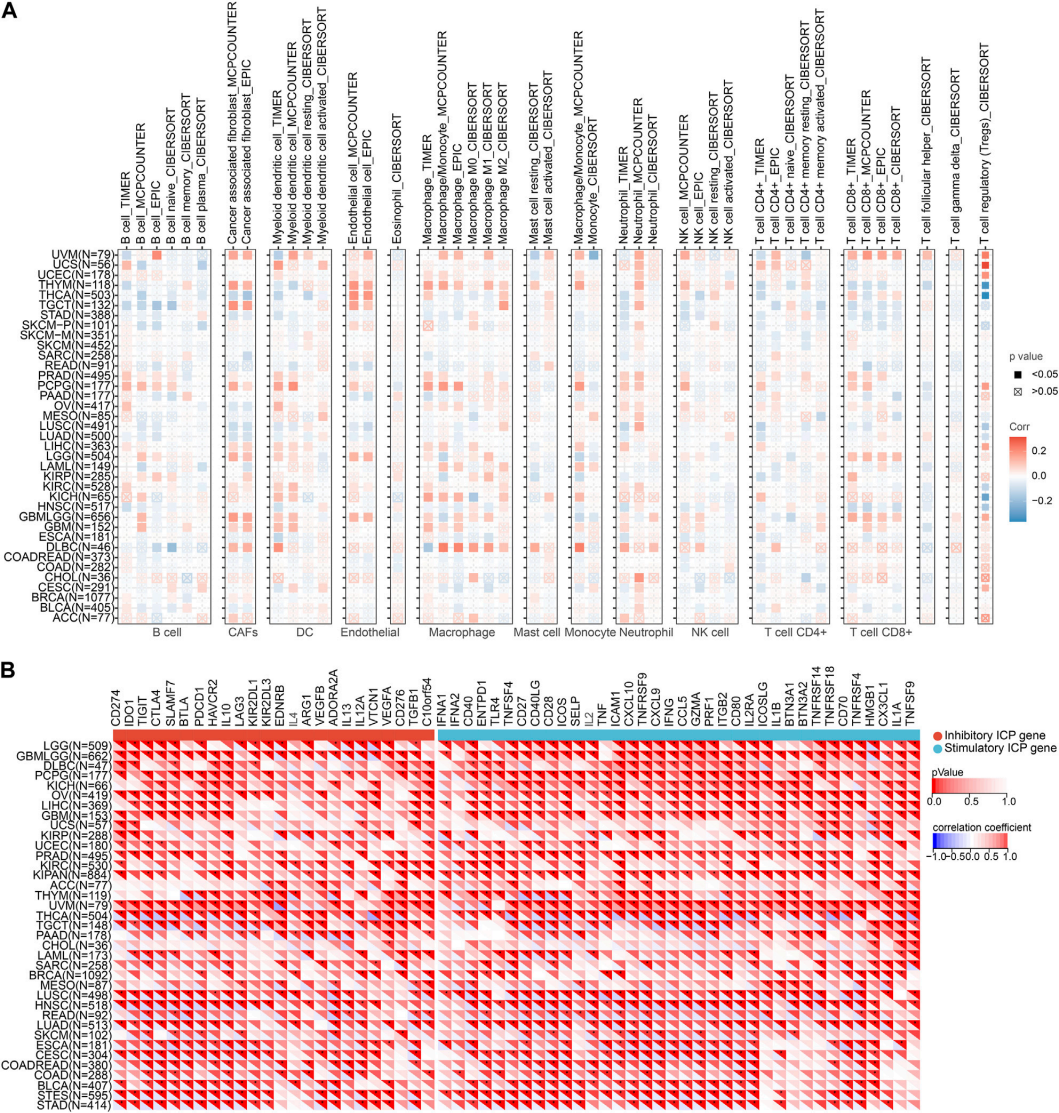
Correlation of *NQO1* mRNA expression immune cell infiltration in pan-cancer. **(A)** Correlations between *NQO1* expression and infiltration of immune cell types were analyzed using the Tumor Immune Estimation Resource (TIMER), the Estimating the Proportion of Immune and Cancer Cells (EPIC), and the Microenvironment Cell Populations-counter (MCP-counter). **(B)** Correlations between *NQO1* expression and immune checkpoint genes for different types of cancer. Blue represents high negative corrections, and red represents high positive corrections; *p < 0.05; **p < 0.01; ***p < 0.001.

Immunotherapy has emerged as a prominent approach in cancer treatment, yet challenges such as limited treatment response and drug resistance persist. In this context, investigating the correlation between *NQO1* and cancer immunity holds great promise as it can offer novel insights and potential breakthroughs for developing more effective immunotherapeutic strategies. Furthermore, tumor cells employ diverse mechanisms to evade the host immune system, which often results in immunotherapy failures. By exploring the intricate relationship between *NQO1* and cancer immunity, we can deepen our understanding of the mechanisms underlying tumor immune escape and potentially unveil new biomarkers and therapeutic targets. Therefore we further explored the *NQO1* correlation with immune cell infiltration in pan-cancer and immune cell infiltration by analyzing the TIMER, EPIC, and MCP counter. The results showed that *NQO1* mRNA expression level is highly co-expressed in several cell types, particularly in dendritic cells (DC), macrophages, and neutrophils. However, the extent to which specific infiltrating cell types correlate with *NQO1* expression in different cancers is inconsistent. Levels of *NQO1* mRNA were negatively correlated with CAFs in lung adenocarcinoma, lung adenocarcinoma, ovarian serous cystadenocarcinoma, stomach adenocarcinoma, and thyroid carcinoma but positively correlated with lymphoid neoplasm diffuse large B-cell lymphoma, glioblastoma multiforme, glioma, brain lower grade glioma, pheochromocytoma, and paraganglioma, testicular germ cell tumors, thymoma, and uveal melanoma. Levels of *NQO1* mRNA were negatively correlated with endothelial in lung squamous cell carcinoma, pancreatic adenocarcinoma, and rectum adenocarcinoma and positively correlated with glioma, brain lower grade glioma, testicular germ cell tumors, thyroid carcinoma, and thymoma (Figure 4A), which illustrates that *NQO1* has different regulatory patterns in various types of cancer. In addition, the correlation of *NQO1* levels of specific cancer with various immune cell subtypes differs. The levels of *NQO1* mRNA were negatively correlated with CD4 T cells and CD8 T cells in testicular germ cell tumors and positively associated with CD4 T cells and CD8 T cells in uveal melanoma and ovarian serous cystadenocarcinoma. In glioma and acute myeloid leukemia, *NQO1* mRNA levels were negatively correlated with CD4 T cells while positively correlated with CD8 T cells (Figure 4A). These findings suggest that heterogeneity and complexity in regulating *NQO1* by different immune cell subtypes in the cancer microenvironment may exist.

Since immune checkpoints genes encoded proteins that regulate the interaction between tumor cells and immune cells on tumor growth and proliferation, we explored the potential role of *NQO1* mRNA expression levels in predicting response to immunotherapy by evaluating the relationship between *NQO1* mRNA expression levels and the 60 immune checkpoints genes in various cancers. The *NQO1* mRNA expression levels were inversely linked with most immunological checkpoint molecules, including thyroid carcinoma, testicular germ cell tumors, lung adenocarcinoma, rectum adenocarcinoma, head and neck squamous cell carcinoma, lung squamous cell carcinoma, stomach adenocarcinoma, stomach and esophageal carcinoma, bladder urothelial carcinoma, colon adenocarcinoma, colon adenocarcinoma/rectum adenocarcinoma esophageal carcinoma, cervical squamous cell carcinoma and endocervical adenocarcinoma, and esophageal carcinoma. These findings suggest that *NQO1* might coordinate the activity of various immune checkpoints genes, including CD274, IDO1, TIGIT, CTLA4, SLAMF7, BTLA, and PDCD1 in different signal pathways, indicating that tumors with high expression levels of *NQO1* could be targeted by immunotherapy (Figure 4B; Supplementary Table S1).

### 3.5 Co-expression of NQO1 in immune and stromal cells

**FIGURE 5:**
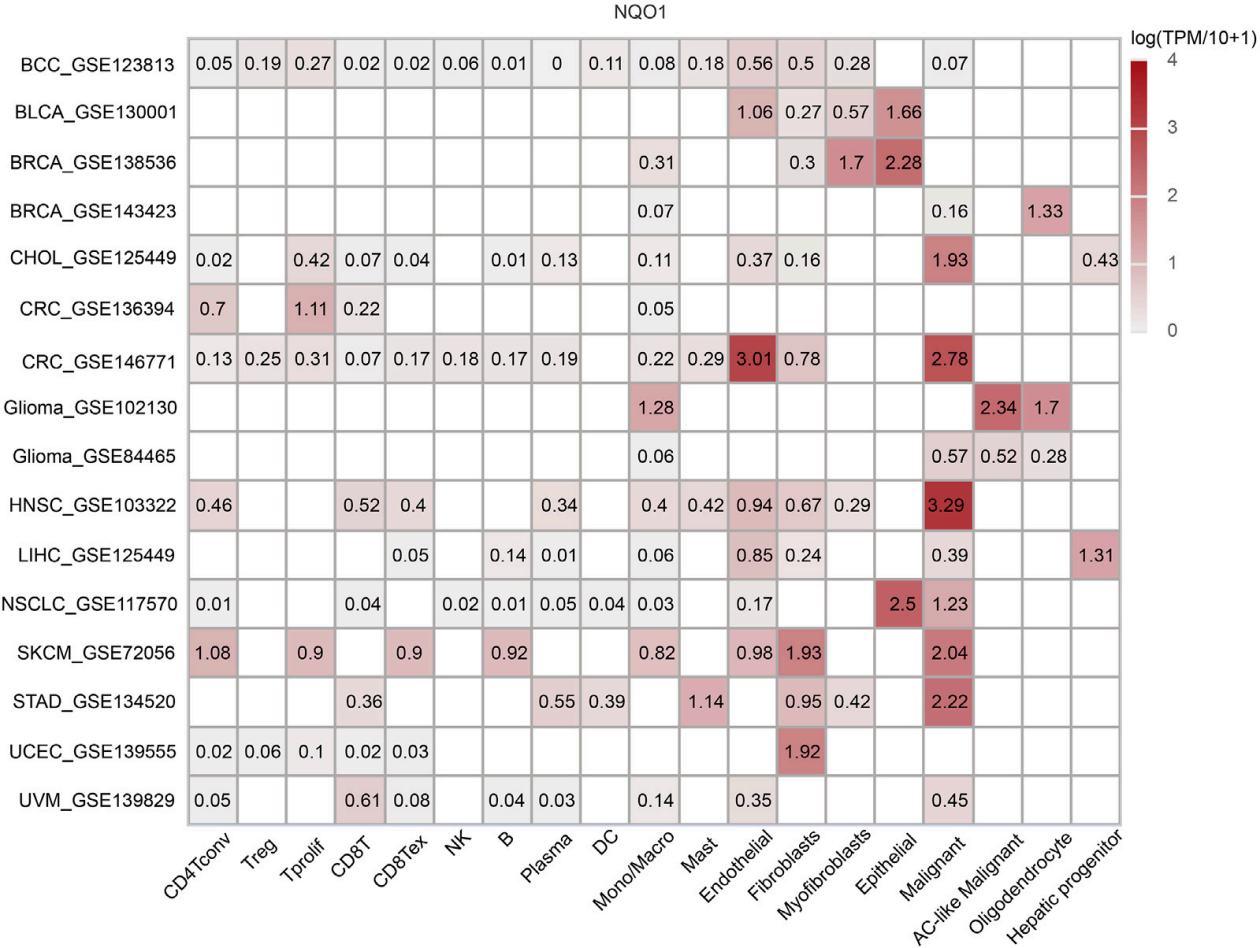
Co-expression of *NQO1* in immune and stromal cells by Single-cell sequencing analysis. The expression levels of *NQO1* mRNA in all cancer cells.

To further explore the association of cell types and *NQO1* in the microenvironment of specific types of cancer cells for study, we analyzed the *NQO1* mRNA expression levels on immune cells in various cancer types using TISCH, 16 single-cell datasets showed relatively higher levels of *NQO1* mRNA. The *NQO1* mRNA was highly expressed in malignant cells in head and neck squamous cell carcinoma, colorectal cancer, skin cutaneous melanoma, stomach adenocarcinoma, and cholangiocarcinoma. *NQO1* was also highly expressed in the epithelial cells in non-small cell lung cancer and invasive breast carcinoma. *NQO1* mRNA was highly expressed in the fibroblast cells of skin cutaneous melanoma, and *NQO1* was highly expressed in endothelial cells in colorectal cancer (Figure 5). We noticed that among the 16 single-cell datasets with high expression of *NQO1*, there were two datasets for invasive breast carcinoma, colorectal carcinoma, and glioma, respectively. Still, there are differences in the results of the two datasets. *NQO1* mRNA was highly expressed in the epithelial and myofibroblasts cells in invasive breast carcinoma (GSE138536), endothelial and malignant cells in colorectal cancer (GSE146771), astrocyte (AC)-like malignant cells in Glioma (GSE102130). However, the results of the invasive breast carcinoma (GSE143423), colorectal cancer (GSE136394), and Glioma (GSE8446) samples differed between them. Upon inquiry, the invasive breast carcinoma (GSE138536) sample was found to be composed of luminal-like and basal-like tumors, but the invasive breast carcinoma (GSE143423) sample was obtained from triple-negative breast cancer patients with brain metastases. Moreover, the molecular characterization of the two samples differed. The ceolorectal cancer (GSE146771) sample was derived from tumors without distant metastasis, while the colorectal cancer (GSE136394) sample was derived from metastatic colorectal cancer tissue with different pathological stages. The Glioma (GSE102130) sample consisted of a K27M mutated tumor, while the glioma (GSE84465) sample did not have this mutation. This suggests that the expression level of *NQO1* mRNA in the microenvironment of specific types of cancer cells may vary according to the cancer origin, histological subtype, pathological stage, and mutation status.

### 3.6 Enrichment analysis and related diseases of *NQO1* gene

**FIGURE 6:**
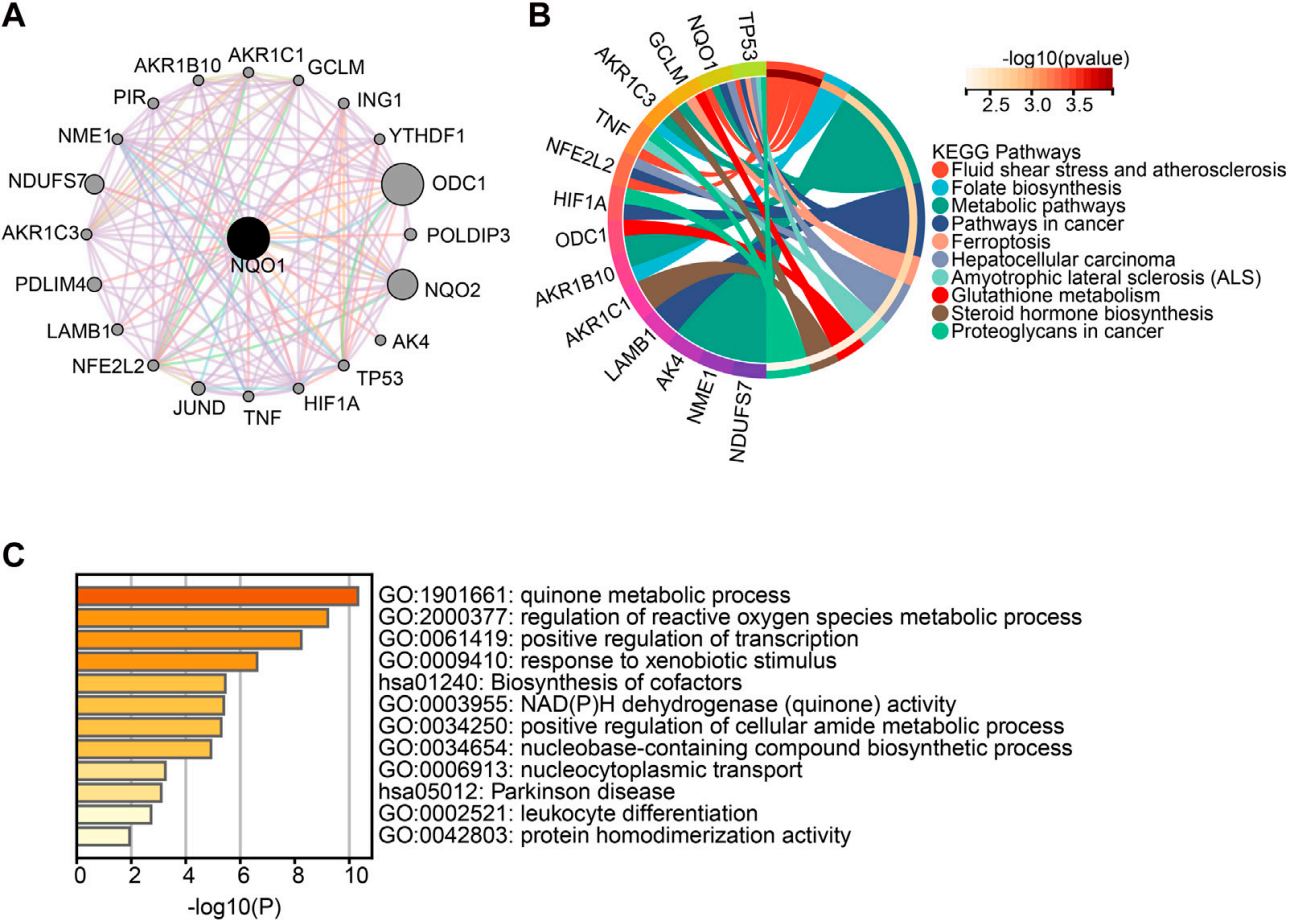
Enrichment analysis of *NQO1*-related gene. **(A)** The network of 20 *NQO1* co-expressed genes was extracted from the STRING tool. **(B)** Kyoto Encyclopedia of Genes and Genomes (KEGG) pathway analysis of the top 20 genes co-expressed with *NQO1*. **(C)** Gene Ontology (GO) analysis of the top 20 genes co-expressed with *NQO1*.

To better understand the mechanisms involved in *NQO1* in tumorigenesis and development, the genes closely related to *NQO1* were obtained from the STRING tool, and the DAVID GO analysis was used to evaluate the *NQO1* biological pathways. A total of 20 genes were found to be co-expressed with *NQO1* (Figure 6A). The Kyoto Encyclopedia of Genes and Genomes (KEGG) pathway analysis showed that *NQO1* co-expressed genes were related to fluid shear and atherosclerosis, folate biosynthesis, metabolic pathways, pathways in cancer, ferroptosis, hepatocellular carcinoma, amyotrophic lateral sclerosis, glutathione metabolism, steroid hormone biosynthesis, proteoglycans in cancer (Figure 6B), where fluid shear stress and atherosclerosis, pathways in cancer, and hepatocellular carcinoma are consistent with *NRF2*-associated gene enrichment pathways (Supplementary Table S2). The GO analysis showed that *NQO1* was closely related to the quinone metabolic process, which is involved in several biological processes, including the regulation of the quinone and the reactive oxygen species metabolic processes, positive regulation of DNA transcription processes, biosynthesis of cofactors, NAD(P)H dehydrogenase (quinone) activity, the cellular amide metabolic process, nucleobase-containing compound biosynthetic process, nucleo-cytoplasmic transport, parkinson disease, leukocyte differentiation, and protein homodimerization activity (Figure 6C). The functional response to the xenobiotic stimulus was consistent with the *NRF2* findings highlighted in Supplementary Table S2. In addition, we also used the DisGeNET database on the Network Analyst website to search for *NQO1*-related diseases. The results demonstrated that several diseases were potentially affected by *NQO1* expression changes, including neoplasms, multiple myeloma, myeloid leukemia, neurotoxicity syndromes, liver disease, alcohol withdrawal syndrome, alcohol intoxication, schizophrenia, bipolar I disorder, mammary neoplasms, prostatic neoplasms, spinocerebellar ataxia, acute kidney injury, mood disorders, occupational diseases, colorectal neoplasms, papilloma, hemolytic-uremic syndrome, Parkinson disease, lymphoma, precursor cell lymphoblastic leukemia, non-small cell lung carcinoma, cholestasis, contact dermatitis, asthma, diabetes mellitus, kidney disease, hemorrhage, and hyperglycemia (Supplementary Figure S4).

### 3.7 Relationship between *NQO1* mRNA expression level with prognosis and anticancer drugs sensitivity

**FIGURE 7:**
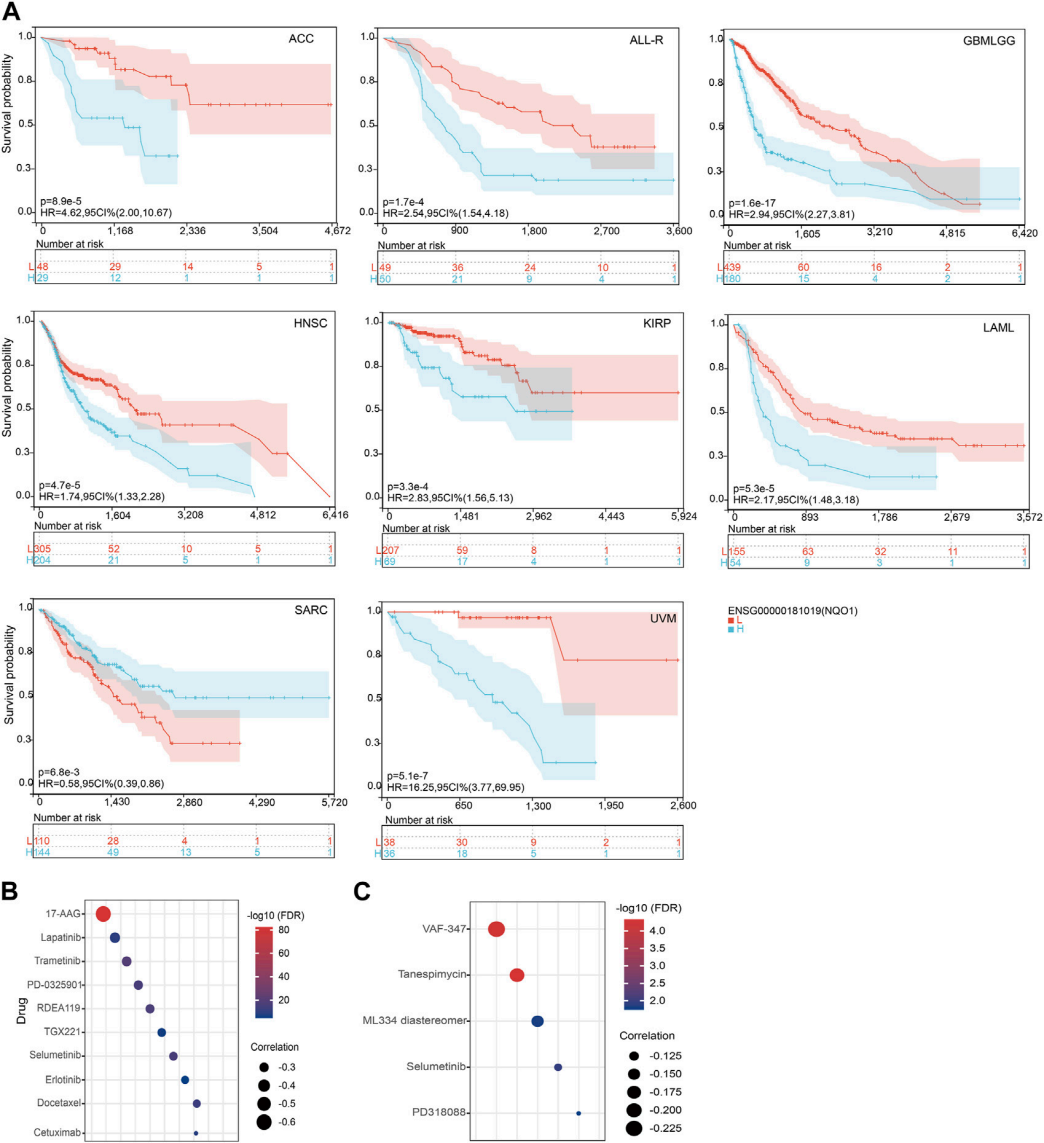
Relationship between *NQO1* mRNA expression level with prognosis and anticancer drus sensitivity. **(A)** K-M plots showed that different *NQO1* levels were associated with overall survival (OS). The Genomics of Drug Sensitivity in Cancer (GDSC) **(B)** and the Cancer Therapeutics Response Portal (CTRP) **(C)** explored the correlation between *NQO1* mRNA expression levels and the half-maximal inhibitory concentration (IC50) for antitumor drugs.

Further, we analyzed the relationship between *NQO1* mRNA expression levels and prognosis in various cancer types through Cox regression analysis. The hazard ratios (HR) for OS were highest for glioma, followed by uveal melanoma, head and neck squamous cell carcinoma, kidney renal papillary cell carcinoma, adrenocortical carcinoma, pan-kidney cohort, brain lower grade glioma, liver hepatocellular carcinoma, acute myeloid leukemia, pancreatic adenocarcinoma, and skin cutaneous melanoma (see Supplementary Figure S5A). Besides, HR for *NQO1* was significantly increased for the PFI of rectum adenocarcinoma and bladder urothelial carcinoma (Supplementary Figure S5B) and DSS of kidney renal clear cell carcinoma (Supplementary Figure S5C). Similar results about disease-free survival are shown (Supplementary Figure S5D). Conversely, high *NQO1* mRNA expression has a protective effect for OS in Sarcoma, PFI, and DSS for prostate adenocarcinoma (Supplementary Figures S5A–C). The subsequent survival analyses, which used patient data dichotomized for optimal cut-off value for adrenocortical carcinoma, acute lymphocytic leukemia, glioma, head and neck squamous cell carcinoma, kidney renal papillary cell carcinoma, kidney renal papillary cell carcinoma, sarcoma, and uveal melanoma (Figure 7A), demonstrate that the OS differences were significant for all cancers, which further confirmed that patients with high *NQO1* mRNA expression had poorer outcomes except for sarcoma (Figure 7A). Similarly, patients with high *NQO1* mRNA expression for PFI of those cancers had poorer outcomes except for prostate adenocarcinoma (Supplementary Figure S6).

To investigate the effect of specific gene expression levels on the sensitivity and resistance to chemotherapeutic drugs, we used the GDSC database to evaluate the correlation between the *NQO1* mRNA expression levels and drug sensitivity (IC50). In this analysis, the *NQO1* mRNA expression levels were negatively correlated with the IC50 of the top 5 drugs: 17-AAG, Lapatinib, Trametinib, PD-0325901, and RDEA119 (Figure 7B; Supplementary Table S3). The CTRP portal evaluated the correlation between the *NQO1* mRNA expression levels and drug resistance. In this evaluation, the *NQO1* mRNA expression levels were negatively correlated with the resistance to the top 5 drugs as VAF-347, Tanespimycin, ML334 diastereomer, Selumetinib, and PD318088 (Figure 7C; Supplementary Table S3). These results suggest that patients with *NQO1* overexpression may respond well to the above drugs and have a low resistance rate. In conclusion, *NQO1* is a potential therapeutic target, and corresponding therapeutic agents or immunotherapies could improve cancer treatment outcomes.

## 4 Discussion

*NQO1* is a ubiquitous soluble enzyme that can be upregulated by the transcription factor *NRF2*, which activates the expression of a variety of genes involved in protecting cells from oxidative damage and, eventually, the development of cancer (Awadallah et al., 2008; Kasai et al., 2016). As a result, several studies evaluated the impact of *NQO1* overexpression on tumor prognosis and its role as a potential target for cancer therapy (Hyun, 2020; Ross and Siegel, 2021). In this study, we performed the first pan-cancer and single-cell analysis to evaluate the impact of the *NQO1* expression on clinicopathological parameters, immune cell infiltration, drug resistance, and prognosis.

Pan-cancer analyses are increasingly being used to evaluate the genetic and molecular differences between various types of cancer to guide the development of new personalized treatment strategies (Ju et al., 2020; Miao et al., 2020). However, since the tumor immune micro-environment may also influence treatment response, single-cell analysis is helpful for the exploration of the tumor immune microenvironment. Hence, we also performed a cluster analysis using single-cell RNA sequencing to assess the role of *NQO1* expression on the tumor immune microenvironment in depth.

In the pan-cancer analysis, we observed the expression of *NQO1* in 31 normal tissues extracted from the GTEx dataset. Consistent with previous work, *NQO1* was mostly expressed in the stomach, thyroid gland, esophagus, adipose tissue, and urinary bladder (Supplementary Figure S1A) (Siegel and Ross, 2000; Yang et al., 2021). In addition, previous studies have also found that *NQO1* is upregulated in many human tumors, and high levels of *NQO1* are related to poorer patient outcomes (Awadallah et al., 2008). Consistent with previous studies, our findings indicate that most cancers had higher expression levels of *NQO1* compared to normal tissue (Figure 1A) (Beaver et al., 2019; Kaghazchi et al., 2022; Yang et al., 2022). The results showed that *NQO1* and *NRF2* were upregulated in acute lymphoeytic leukemia, cervical squamous cell carcinoma and endocervical adenocarcinoma, cholangiocarcinoma, cholangiocarcinoma, glioblastoma multiforme, glioma, acute myeloid leukemia, brain lower grade glioma, brain lower grade glioma, pancreatic adenocarcinoma, stomach adenocarcinoma, stomach and esophageal carcinoma, stomach and esophageal carcinoma, kidney renal clear cell carcinoma, pan-kidney cohort, kidney renal papillary cell carcinoma, and wilms tumor, suggesting that *NQO1* may be involved in the process of tumor development and that the transcription factor *NRF2* regulated its expression level. In addition, the upregulation of *NQO1* and downregulation of *NRF2* in bladder urothelial carcinoma, lung adenocarcinoma, ovarian serous cystadenocarcinoma, prostate adenocarcinoma, skin cutaneous melanoma, testicular germ cell tumors, uterine corpus endometrial carcinoma, and uterine carcinosarcoma, indicatesthe inconsistent direction of changes observed between the *NQO1* and *NRF2* expression level may be related to differences in the methylation levels (Supplementary Figure S2B). These differences may reflect the diverse mechanisms regulating *NQO1* expression in different cancer types.

Multiple factors contribute to variations in the risk of cancer development and progression between males and females. The prevalence of unhealthy lifestyle choices associated with cancer development (especially smoking) varies significantly between genders. Moreover, sex hormones can also affect metabolism, immunity, inflammation, and, ultimately, the fidelity of the genetic code. Thyroid carcinoma, dead and neck squamous cell carcinoma, lung squamous cell carcinoma, lung adenocarcinoma, liver hepatocellular carcinoma, bladder urothelial carcinoma, kidney renal papillary cell carcinoma, and kidney renal clear cell carcinoma have broader sex-biased molecular labels. These cancers have shown higher incidences of gender-related differences in molecular features and mortality rates. (Ross and Siegel, 2021). In addition, the National Comprehensive Cancer Network (NCCN) Clinical Practice Guidelines in Oncology identified gender as a prognostic indicator for five cancers (i.e., lung squamous cell carcinoma, lung adenocarcinoma, head and neck squamous cell carcinoma, kidney renal clear cell carcinoma, and kidney renal papillary cell carcinoma) (Yuan et al., 2016). However, the mRNA expression analysis in the study revealed fewer gender-biased genes within normal tissue samples, suggesting that gender bias may be amplified during tumorigenesis (Yuan et al., 2016). These findings highlight the need to analyze the impact of gender on cancer initiation, invasion, and the development of drug resistance. Furthermore, estrogen can reduce mitochondrial damage and the production of reactive oxygen species in women (Haupt et al., 2021). These estrogen-mediated factors can enhance the effects of cancer therapy in women by reducing the activation of the *NQO1* transcription factor *NRF2* and regulating other antioxidant-related transcription factors via the NRF2 pathway (Kim, 2022). Our results show that *NQO1* differs between genders in lung adenocarcinoma, lung squamous cell carcinoma, liver hepatocellular carcinoma, lymphoid neoplasm diffuse large B-cell lymphoma, kidney renal papillary cell carcinoma, and sarcoma. The results in this study suggest that *NQO1* may be associated with disease onset and progression in patients with sex-specific cancers. Therefore further research is required to evaluate the impact of *NQO1* in specific genetic gender factors in the treatment of cancer patients.

*NQO1* is highly upregulated in carcinogenesis, which makes it an ideal diagnostic and prognosis biomarker (Awadallah et al., 2008; Hirose et al., 2021; Zhu et al., 2020). Our study conducted Cox regression analysis, which revealed that cancers with high expression of *NQO1*, including glioma, uveal melanoma, head and neck squamous cell carcinoma, kidney renal papillary cell carcinoma, adrenocortical carcinoma, and pan-kidney cohort, had significantly higher hazard ratios for OS (Supplementary Figure S5A). The underlying mechanism of *NQO1* in tumorigenesis remains is still unclear, upregulation of *NQO1* is thought to help cancer cells deal with elevated oxidative stress (Madajewski et al., 2016).

NQO1 is shown to be a protective and multifunctional antioxidant that regulates oxidative stress and DNA damage in chromatin-binding proteins in cancer cells (Preethi et al., 2022). Meanwhile, the *NQO1*’s ability to bind to proteins is influenced by the acidification of intracellular pyridine nucleotides (Ross and Siegel, 2021). NQO1 is involved in various carcinogenic processes, such as the detoxification of quinone scavenger of superoxide anion radical and antioxidant enzymes, as well as the stabilization of proteins.GO analysis showed a close association between the quinone metabolic process responsible for the production of ROS as part of the metabolic cell process and the transcription of the RNA polymerase Ⅱ in response to hypoxia. This is aligns with previous studies. Additionly, *NQO1* and *NRF2* are known to be associated with ferroptosis in tumors (Liu et al., 2022; Shan et al., 2020; Zheng et al., 2022), neurodegenerative diseases (Yang et al., 2022), amyotrophic lateral sclerosis (Peng et al., 2022). In addition, previous studies found that the alteration of the NRF2/NQO1 pathway in rat testicular tissue could affect sperm and steroid production (Shi and Fu, 2019). NRF2 and NQO1 are also involved in the regulation of the cellular amide metabolic process, which regulates amide damage to liver function (Gao et al., 2023; Liu et al., 2021). In addition, we also found that NQO1 expression was significantly associated with glutathione metabolism and that this pathway is used as the main regulatory target by NRF2. Genes transcribed by NRF2 facilitate the synthesis of glutamine (Mitsuishi et al., 2012). In addition, NQO1 was involved in leukocyte differentiation via the regulation of reactive oxygen species levels in various immune cells, such as in the T helper 17 cells (Nishida-Tamehiro et al., 2022). These pathways were supported by our findings. In addition, our analysis found that NQO1 and NRF2 are involved in response to xenobiotic stimulus, fluid shear stress, and atherosclerosis pathways in cancer. Previous studies have shown that oxidative stress-related genes such as NQO1 are enriched in fluid shear stress and atherosclerotic pathways in a hydrogen peroxide-induced RIN-m5F cell injury model, suggesting that the NRF2 signaling pathway may play a protective role (Zhou et al., 2023). However, our study also found that NQO1 may also be related to folate biosynthesis and response to the xenobiotic stimulus. Folate biosynthesis promotes NADPH production (Fan et al., 2014; Girish et al., 2022; Pathikkal et al., 2022). Thus, NQO1 may use the electrons provided by NADPH during folate metabolism to participate in the reduction reaction of substrates.This reaction helps to reduce oxidative stress and protects cells from oxidative damage.Indeed, NQO1 also known to act on various exogenous substates, particularly xenobiotic quninones (Ross and Siegel, 2021). This characteristic provides a plausible explanation for the association between NQO1 and the response to xenobiotic stimuli. In addition, due to its essential role in redox processes, *NQO1* was associated with other diseases, such as multiple sclerosis, Alzheimer’s disease (Beaver et al., 2019), type 2 diabetes, and metabolic syndrome (Ross and Siegel, 2021). Our results have also shown that *NQO1* is also associated with psychiatric disorders, hematological disorders, liver disease, and kidney injury.

The immune system has an important role in cancer progression (Dobosz and Dzieciatkowski, 2019). Several immunotherapy drugs have been developed to stimulate the immune system to fight cancer (Esfahani et al., 2020; Yang et al., 2022). Immune checkpoint inhibitors can block the immune system from attacking the cancer cells and are increasingly being used to treat this disease (Liang et al., 2021; Wang et al., 2021; Zhang et al., 2021). Tumor-infiltrating immune cells are crucial in cancer progression, invasion, and drug resistance (Galli et al., 2020; Hiam-Galvez et al., 2021; Zhang et al., 2021; Zhang et al., 2021; Zhang et al., 2022). The effect of *NQO1* on the tumor immune microenvironment was not thoroughly studied. In this study, we systematically analyzed the role of *NQO1* as a novel immunotherapeutic target in 33 cancers and found that *NQO1* was closely related to immune infiltration. *NQO1* was highly expressed in cancer cells and several cells, especially DC, macrophages, neutrophils, and T cells. This implies that *NQO1* may play an important role in regulating the immune response of tumors. Understanding the role of *NQO1* in the tumor immune microenvironment will help us better understand the mechanisms of tumor immune escape and provide guidance for the development of new immunotherapeutic strategies. However, this correlation differs in specific tumors or specific cell subtypes, suggesting that the expression and regulatory patterns of *NQO1* levels vary in different cancer types and immune cell subtypes. These findings suggest that the role of *NQO1* in different cell subtypes needs to be considered when designing novel immunotherapies. Individualized treatment regimens must be developed for each cancer type, target their specific T-cell subtypes and differences in *NQO1* levels (Figure 4A). The further single-cell analysis also showed higher expression levels of *NQO1* in the stromal cells, epithelial, fibroblasts, and endothelial cells in various cancers, particularly bladder urothelial carcinoma, invasive breast carcinoma, colorectal cancer, and glioma (Figure 5). Thus, the high expression of *NQO1* in these cell types suggests its role in regulating tumor development by *NQO1*, possibly by influencing the extracellular matrix formation of these cells and the proliferation and extension of tumor cells. However, the *NQO1* mRNA expression levels in our study varied between the same tumor types with different molecular phenotypes, pathological stages, or mutation types. These findings suggest that the diversity of study subjects must be considered when studying gene expression to improve the understanding of their clinical features and biological significance, as shown in Figures 2A, B. In addition, we also found that *NQO1* is positively correlated with the tumor mutation burden in 7 cancer types and positively related to microsatellite instability in 5 cancer types, indicating *NQO1* expression may affect the tumor mutation burden and microsatellite instability levels in different cancers and immuno-therapy responses (Figures 3A, B). It is suggested that *NQO1* expression may affect the level of tumor mutation burden and microsatellite instability in these tumors, and patients with tumors with high *NQO1* expression may be more sensitive to immunotherapy. Understanding the relationship between *NQO1* and the tumor mutation burden and microsatellite instability can provide new insights for the development of individualized cancer treatment, thus improving the efficacy of cancer therapy. For example, radiation-resistant tumors of early-stage laryngeal squamous cell carcinoma patients had increased the tumor mutation burden and *NQO1* expression (Sheth et al., 2021).

Tumors have different levels of *NQO1* compared to normal tissue, and compounds containing *NQO1*-activated quinone pharmacophore should have significant tumor selectivity, which may lead to the development of *NQO1*-directed antitumor agents (Ross and Siegel, 2021). Thus, *NQO1* is a worthwhile target for personalized anticancer therapy (Beaver et al., 2019; Ross and Siegel, 2021; Starcher et al., 2020). For example, studies have shown that targeting *NQO1* can effectively trigger innate sensing within the tumor microenvironment, synergizing with immunotherapy to overcome adaptive drug resistance (Li et al., 2019). Public databases and computational models are often used to identify individualized drug therapy (Adam et al., 2020; Rydzewski et al., 2021; Zhang et al., 2022). In this paper, we evaluated the impact of different *NQO1* expression levels on the sensitivity of several chemotherapy drugs using data extracted from two public databases. In this analysis, we found that the *NQO1* expression levels were negatively correlated with IC50 of 17-AAG, Lapatinib, Trametinib, PD-0325901, and RDEA119 (Figures 7B, C). The anticancer agent 17-AAG belongs to the class of heat shock protein 90 (Hsp90) inhibitors. Numerous studies have shown that *NQO1* plays a crucial role in enhancing the antitumor efficacy of 17-AAG as it acts as a pivotal enzyme for its bioactivation (Kasai et al., 2016). For example, in the esophageal squamous cell carcinoma cell lines, the sensitivity of cancer cells to 17-AAG was significantly positively correlated with the *NQO1* expression levels (Hadley and Hendricks, 2014). Conversely, in melanoma and non-small cell lung cancer cell lines, the *NQO1* expression levels were negatively correlated with sensitivity to 17-AAG (Kasai et al., 2016). In the viability screening assays, treatment of tumor cell lines using maximal concentrations induced the production of reactive oxygen species, while the tyrosine kinase inhibitor analogs, such as Lapatinib, had the strongest ability to induce oxidative stress (Akhtari et al., 2021). Lapatinib is a tyrosine kinase inhibitor that can inhibit tumor survival and proliferation by blocking the activation of HER1 and HER2 tyrosine kinases and the subsequent activation of downstream pathways. Previous Studies have shown that Lapatinib can inhibit tumor cell proliferation in breast cancer by increasing *NQO1* expression (Zhang et al., 2020). These findings suggest that *NQO1* may improve the efficacy of these drugs in certain cancers. Trametinib (Zhao et al., 2021), PD-0325901 (Mirdametinib) (van Geel et al., 2020), and RDEA119 (Refametinib) (Dilly et al., 2015) belong to the mitogen-activated protein kinase inhibitors (MEK) class. These compounds exert their antitumor effects by inhibiting the growth and proliferation of tumor cells via the suppression of MEK enzyme activity and the subsequent blockade of their associated signaling pathways. However, there is a current lack of reported studies evaluating the therapeutic impact of modulating *NQO1* with these drugs.In summary, these findings confirmed that the expression levels of *NQO1* could be used to predict the response to immunotherapy and small-molecule drugs. Patients with *NQO1* overexpression may respond well to the above drugs and have a low resistance rate. However, the specific mechanisms of action of these drugs and their importance in the antitumor effects need further studies to be elucidated.

However, even though we performed a comprehensive and systematic analysis of *NQO1*, there are limitations in our study. First, experiments *in vivo* and *in vitro* are needed to verify the potential relationship between *NQO1* with immune infiltration and the prognosis of cancers. Second, we have no specific data to determine the role of *NQO1* in antitumor immunotherapy. Therefore, more experimental work is needed to identify *NQO1* function in cancer in further study. Moreover, due to the high heterogeneity and limited availability of hematological cancer tissue samples within the TCGA database, we could not perform an in-depth exploration to evaluate the impact of *NQO1* on all hematologic tumor subtypes. Therefore further research is required to explore the impact of *NQO1* on hematological malignancies.

## 5 Conclusion

The present study systematically explored the expression levels and prognostic significance of NQO1 and the relationship of NQO1 with clinicopathological parameters and immune cell infiltration by pan-cancer and single-cell analysis. We conclude that NQO1 significantly correlates with prognosis and immune infiltrates in multiple cancer types. The NQO1 expression levels could also predict the response to several immunotherapies. Developing immunotherapies that inhibit the NQO1-dependent signaling pathways may provide a promising approach to cancer treatment.

## Data Availability

The original contributions presented in the study are included in the article/Supplementary Material, further inquiries can be directed to the corresponding authors.

## Supplementary material

SUPPLEMENTARY FIGURE S1 Prognostic analysis of *NQO1* mRNA expression in pan-cancer. **(A)** NQO1 mRNA expression level in the 31 normal human tissues extracted from the GTEx dataset. The NQO1 mRNA levels in the 32 tumor cell lines were extracted from the CCLE database **(B)** and 69 from the HPA dataset **(C)**.

SUPPLEMENTARY FIGURE S2 Expression levels of *NRF2* mRNA and methylation levels of *NRF2* and *NQO1*. **(A)** The *NRF2* mRNA expression levels in cancerous (blue bar) and normal (red bar) tissues according to the data extracted from the Cancer Genome Atlas (TCGA) and the Genotype-Tissue Expression (GTEx) databases. **(B)** Analysis of NQO1 and NRF2 methylation levels. **(C)** *NQO1* mRNA expression level beteewn gender in the control group.

SUPPLEMENTARY FIGURE S3 Comparison of the NQO1 protein expression levels between normal and cancerous tissue including; **(A)** colon, liver, lung, and prostate; **(B)** Pan-cancer analyses evaluating the association between the *NQO1* mRNA expression levels according to age for cancers.

SUPPLEMENTARY FIGURE S4 Diseases that may be affected by *NQO1* expression changes as predicted by the DisGeNET database on the Network Analyst website.

SUPPLEMENTARY FIGURE S5 Association of *NQO1* mRNA expression level with overall survival (OS) **(A)**, patient progression-free interval (PFI) **(B)**, patient disease-specific survival (DSS) **(C)**, and patient disease-free survival (DFS) **(D)**.

SUPPLEMENTARY FIGURE S6 K-M plots showed that different *NQO1* mRNA levels were associated with progression-free interval (PFI).

SUPPLEMENTARY TABLE S1 Correlation of *NQO1* mRNA expression with immune checkpoint-related genes.

SUPPLEMENTARY TABLE S2 Go and Pathway analysis of NRF2.

SUPPLEMENTARY TABLE S3 Correlation between the sensitivity to chemotherapy (the half-maximal inhibitory concentration, IC50) and *NQO1* mRNA expression levels.
