# Supplementary figures and images for: Pan-cancer and single-cell analysis reveal the prognostic value and immune response of NQO1
Source: Front Cell Dev Biol. 2023 Jul 31;11:1174535. doi: 10.3389/fcell.2023.1174535 (PMC10424457; doi:10.3389/fcell.2023.1174535)

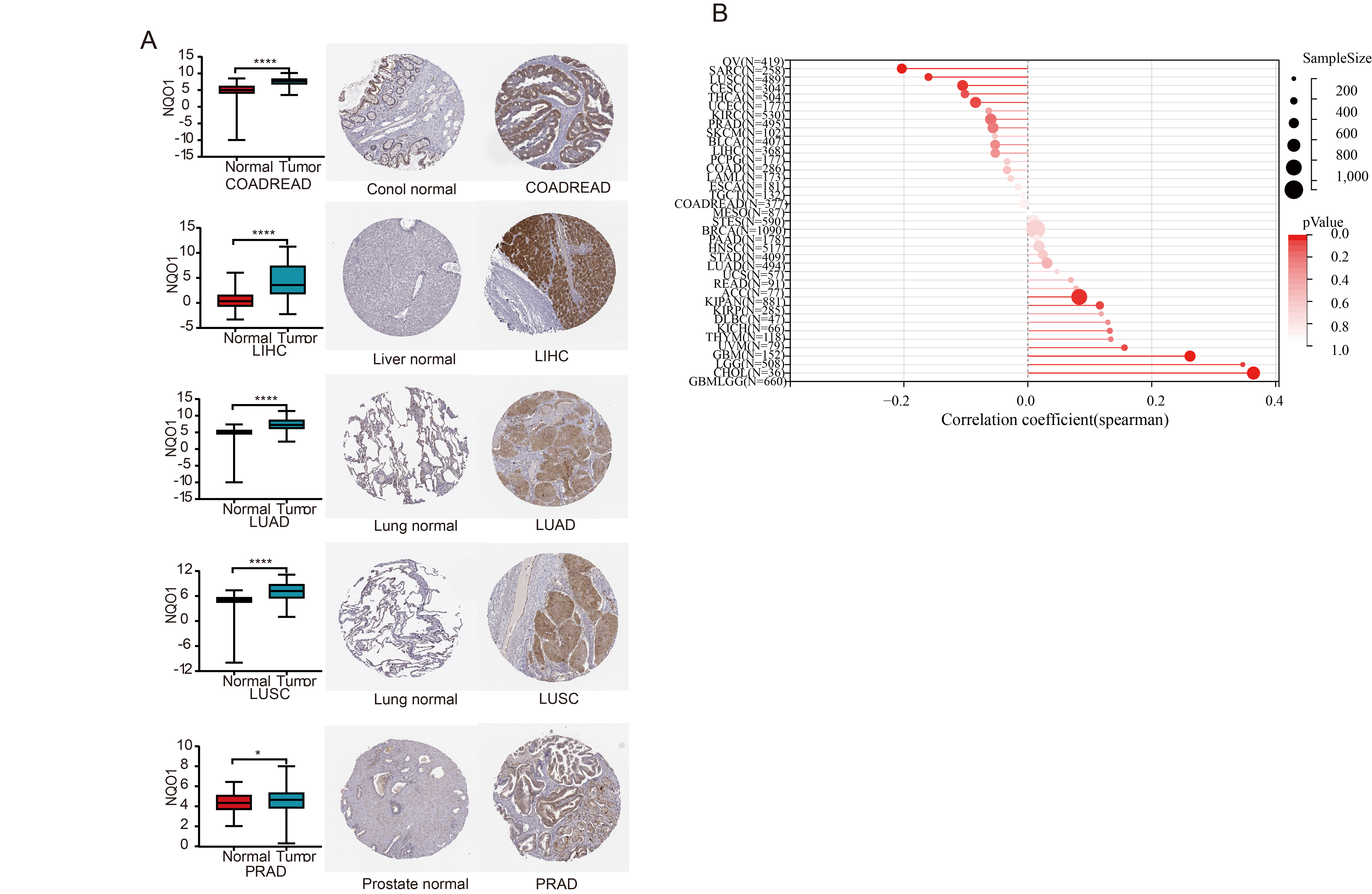

Supplement: Supplementary file 2 [file Image3.JPEG]

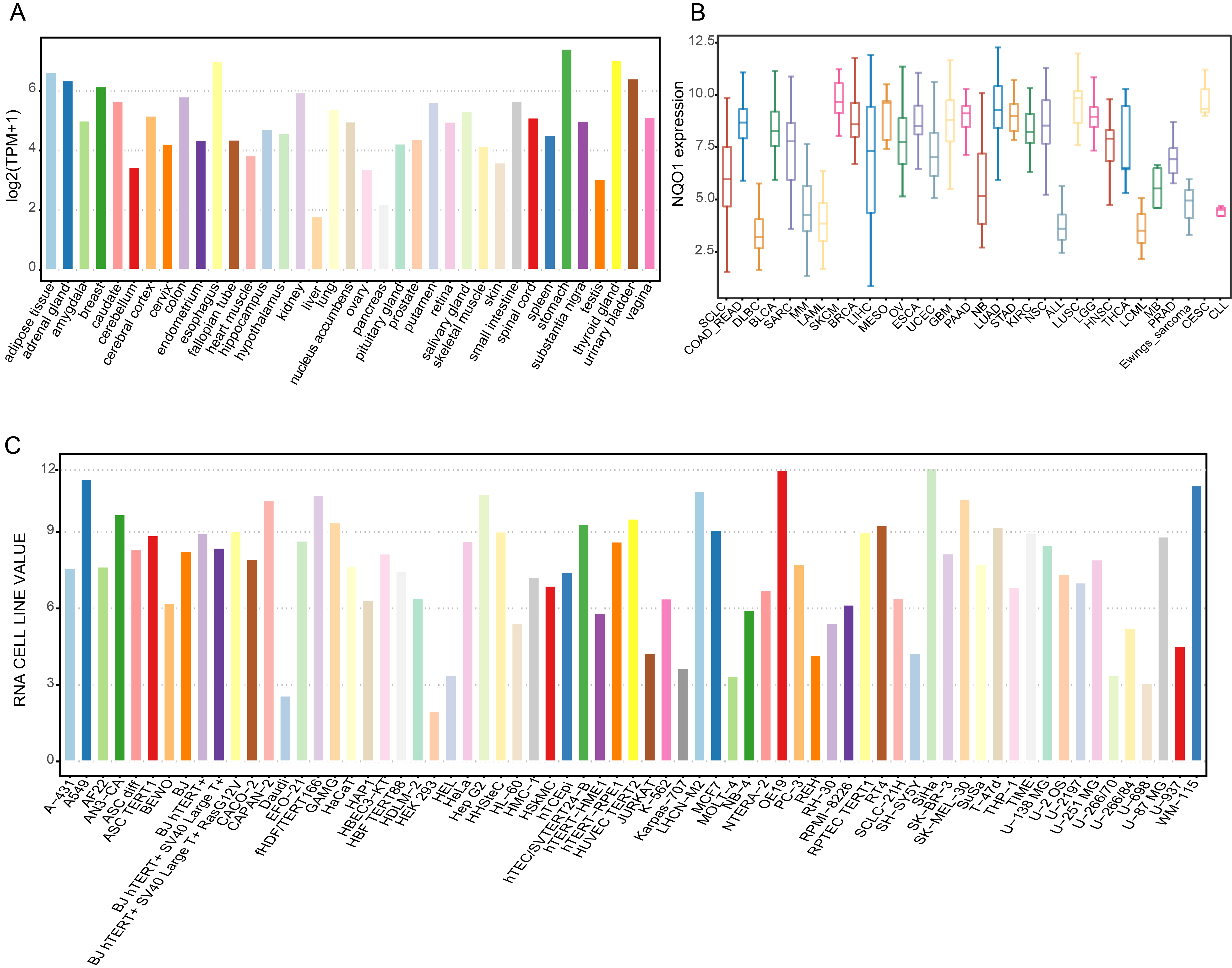

Supplement: Supplementary file 4 [file Image1.JPEG]

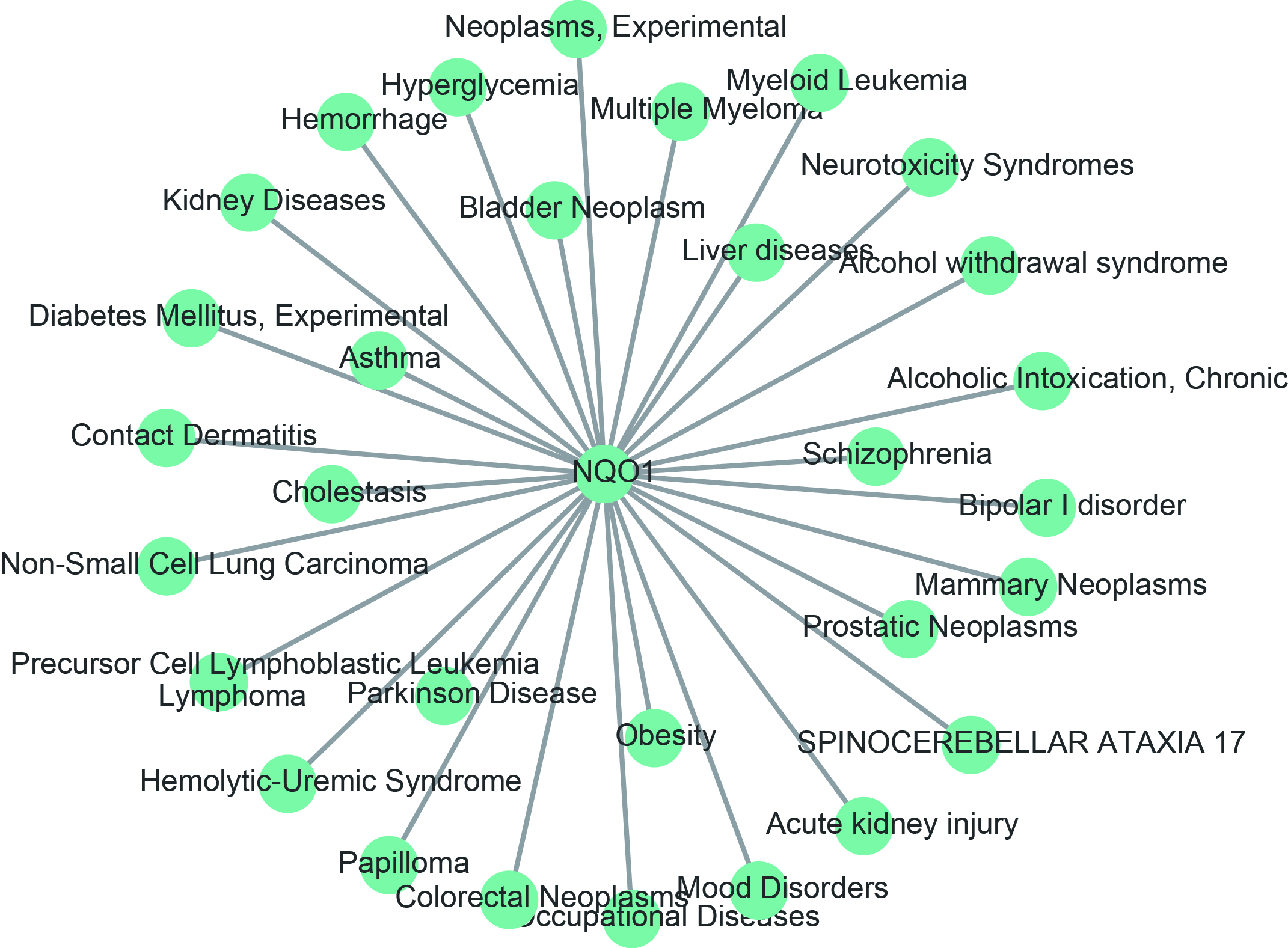

Supplement: Supplementary file 5 [file Image4.JPEG]

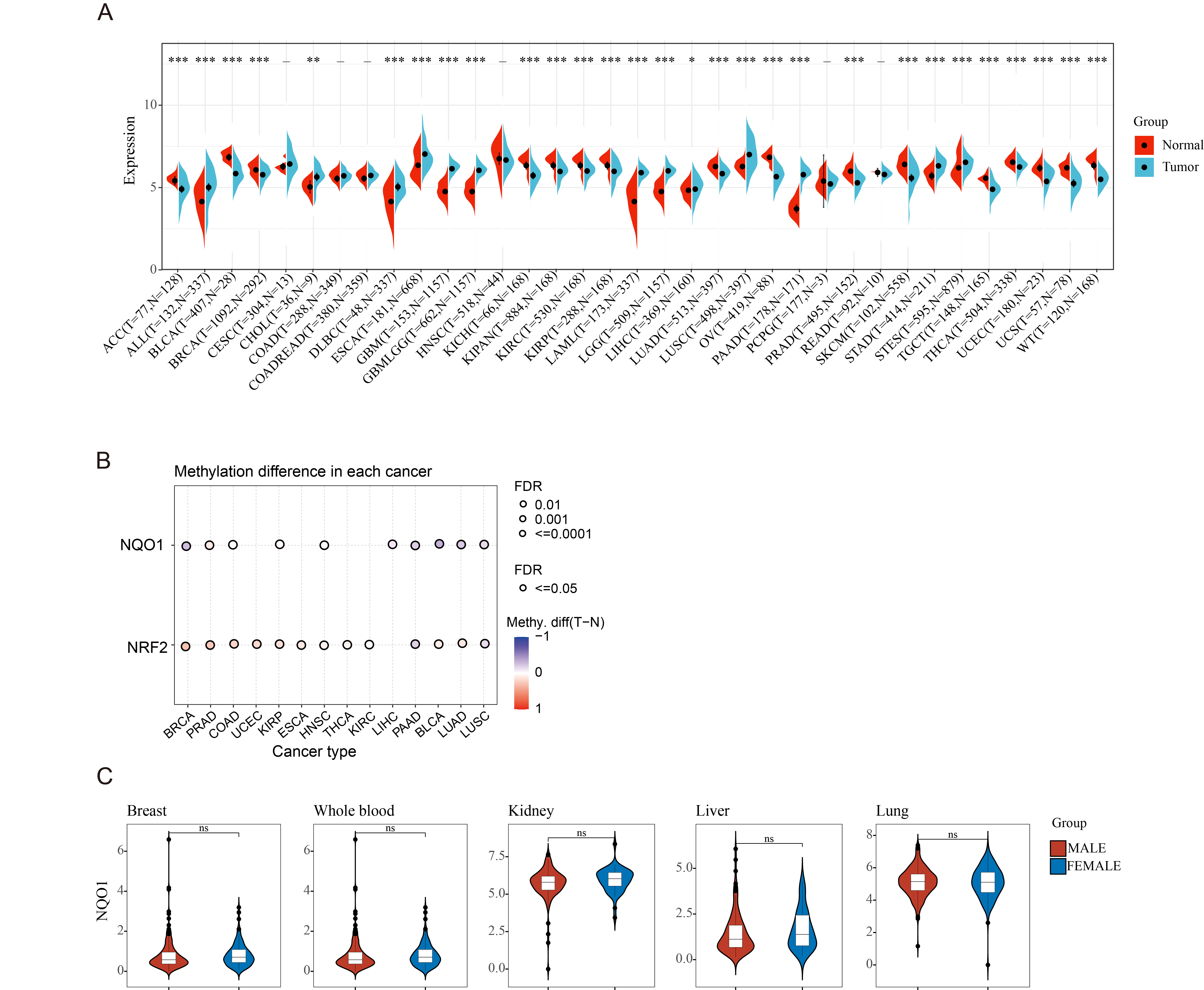

Supplement: Supplementary file 6 [file Image2.JPEG]

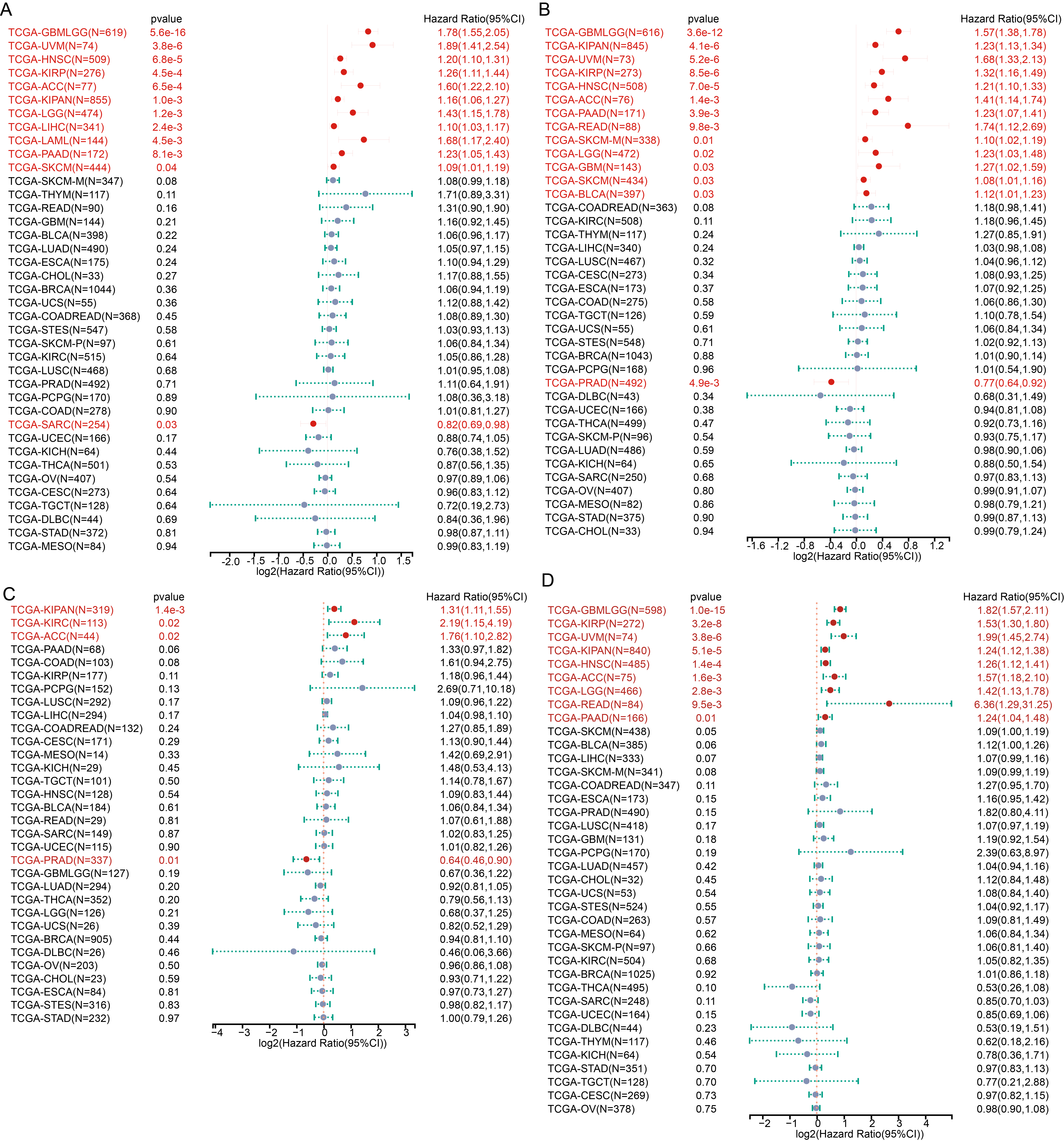

Supplement: Supplementary file 7 [file Image5.JPEG]

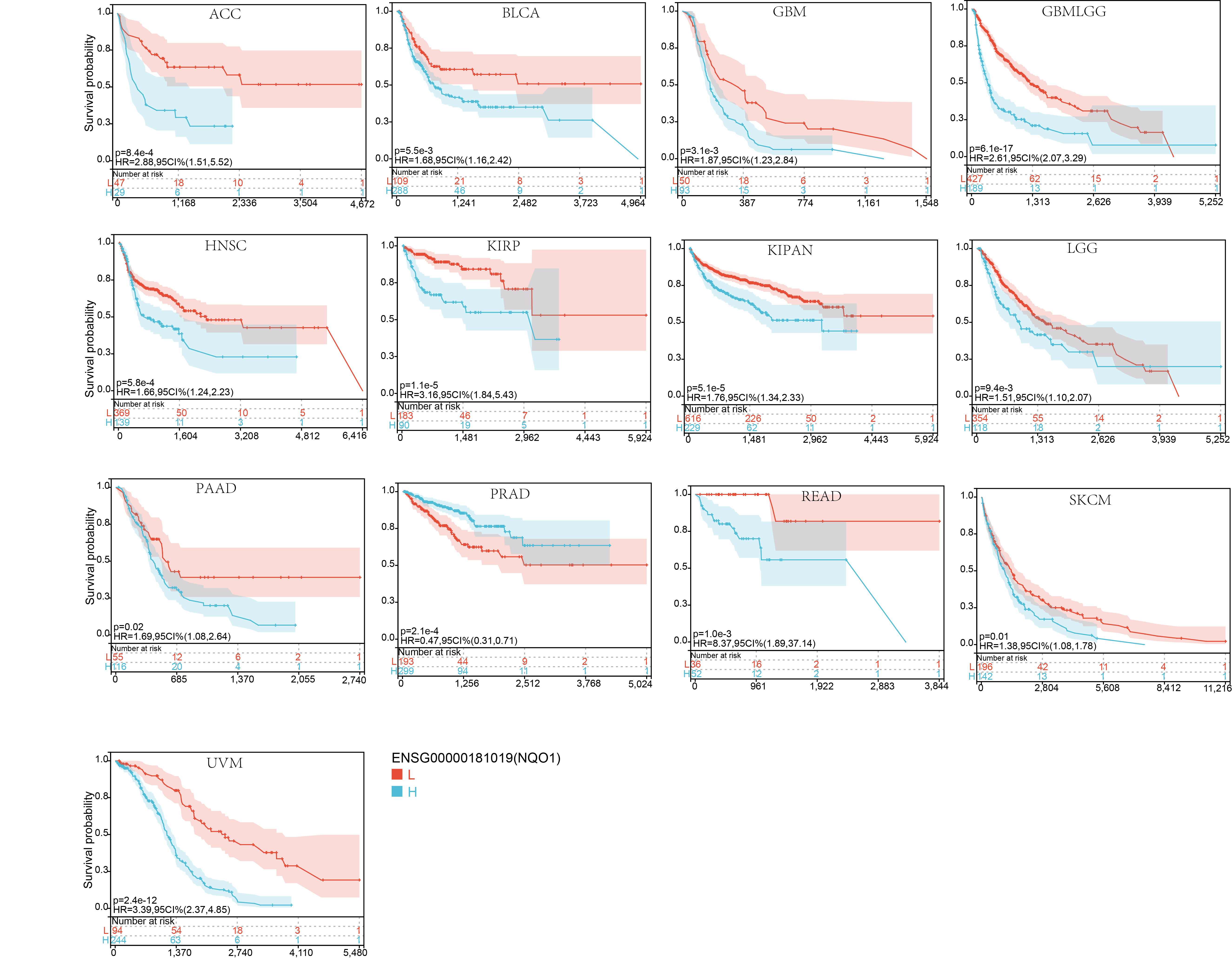

Supplement: Supplementary file 9 [file Image6.JPEG]
